# Post-cardiac injury syndrome in acute myocardial infarction patients undergoing PCI: a case report and literature review

**DOI:** 10.1186/s12872-018-0964-4

**Published:** 2018-12-12

**Authors:** Yan Gao, Nanette H. Bishopric, Hong-wei Chen, Jiang-tao Li, Yu-lang Huang, He-xun Huang

**Affiliations:** 1 Department of Cardiovascular Medicine, Shenzhen Shekou People’s Hospital, Shenzhen, 518067 Guangdong China; 2 Departments of Medicine, Molecular and Cellular Pharmacology and Pediatrics, University of Miami Miller School of Medicine, Miami, FL USA; 3 Department of Interventional Diagnosis and Treatment, Shenzhen Longhua Central Hospital, Shenzhen, 518067 Guangdong China

**Keywords:** Post-cardiac injury syndrome, Acute myocardial infarction, Primary percutaneous coronary intervention, Coronary microvascular dysfunction

## Abstract

**Background:**

In the era of primary percutaneous coronary intervention (PPCI), the incidence of post-cardiac injury syndrome (PCIS) in patients with acute myocardial infarction (AMI) following PPCI has become less common. However, the intrinsic pathogenesis of this medical condition remains largely uncertain. Unlike the prior reports, the present paper provides new mechanistic clues concerning the pathogenesis of PCI-related PCIS.

**Case presentation:**

A 45-year-old male with AMI had developed an early onset of PCIS at 3 h after PPCI. A significantly slower TIMI flow (grade ≤ 2) for the culprit arteries was observed through follow-up coronary angiography (CAG); no stent thrombosis or any significant evidence of iatrogenic trauma due the intervention procedures was found. Nevertheless, the the serum level of HsCRP showed similar variation trend as the neutrophil count and troponin T in continuous blood monitoring, which suggested a potential association between PPCI-related coronary microvascular dysfunction (CMD) and pathogenesis of PCIS.

**Conclusions:**

The reported case had excessive inflammatory reaction and CMD resulting from cardiac ischemia-reperfusion injury in an AMI patient with risk factors of endothelial dysfunction. There exists a potential reciprocal causation between PCIS and performance of PPCI in the AMI patient who was susceptible to endothelial damage.

## Background

Post-cardiac injury syndrome (PCIS) is referred to as an autoimmune reaction resulting from a variety of cardiac insults, including myocardial necrosis, cardiac trauma and surgery. In the era of primary percutaneous coronary intervention (PPCI), the incidence of PCI-related PCIS has been reported as lower than 0.5%, while its intrinsic pathogenesis remains completely uncertain [1]. Here we report an unusual case of early-onset PCIS after PPCI for acute myocardial infarction (AMI) plus post-operative coronary microvascular dysfunction (CMD). We further discuss the possible pathogenesis and risk alleviation of PCI-related PCIS.

## Case presentation

**Fig. 1:**
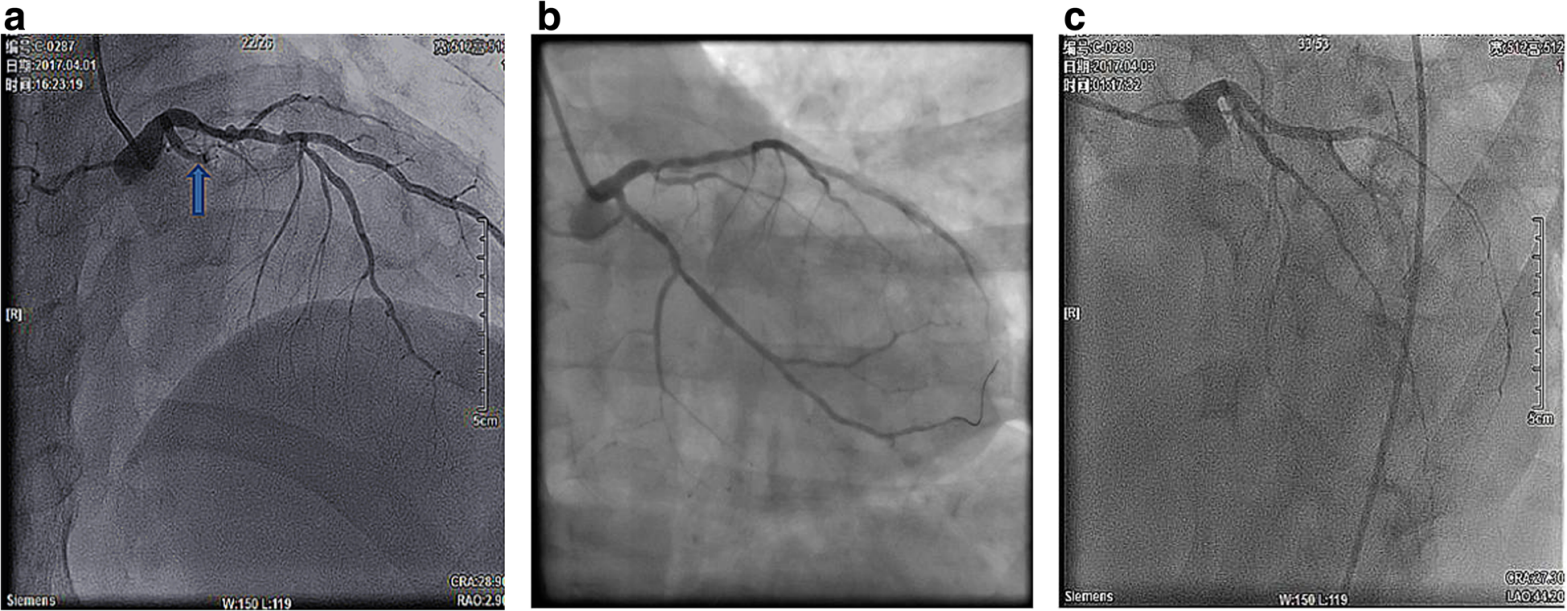
**a** Pre-intervention image: coronary angiogram showed complete proximal occlusion of the circumflex artery (arrow). **b** Post-intervention image: A drug eluting stent was successfully deployed to the proximal circumflex artery (p-LCX), and the final angiogram showed optimal result. **c** Follow-up coronary angiography image after 33 h of PCI showed no stent thrombosis but significant slower TIMI flow (grade ≤ 2 grade) than before

**Fig. 2:**
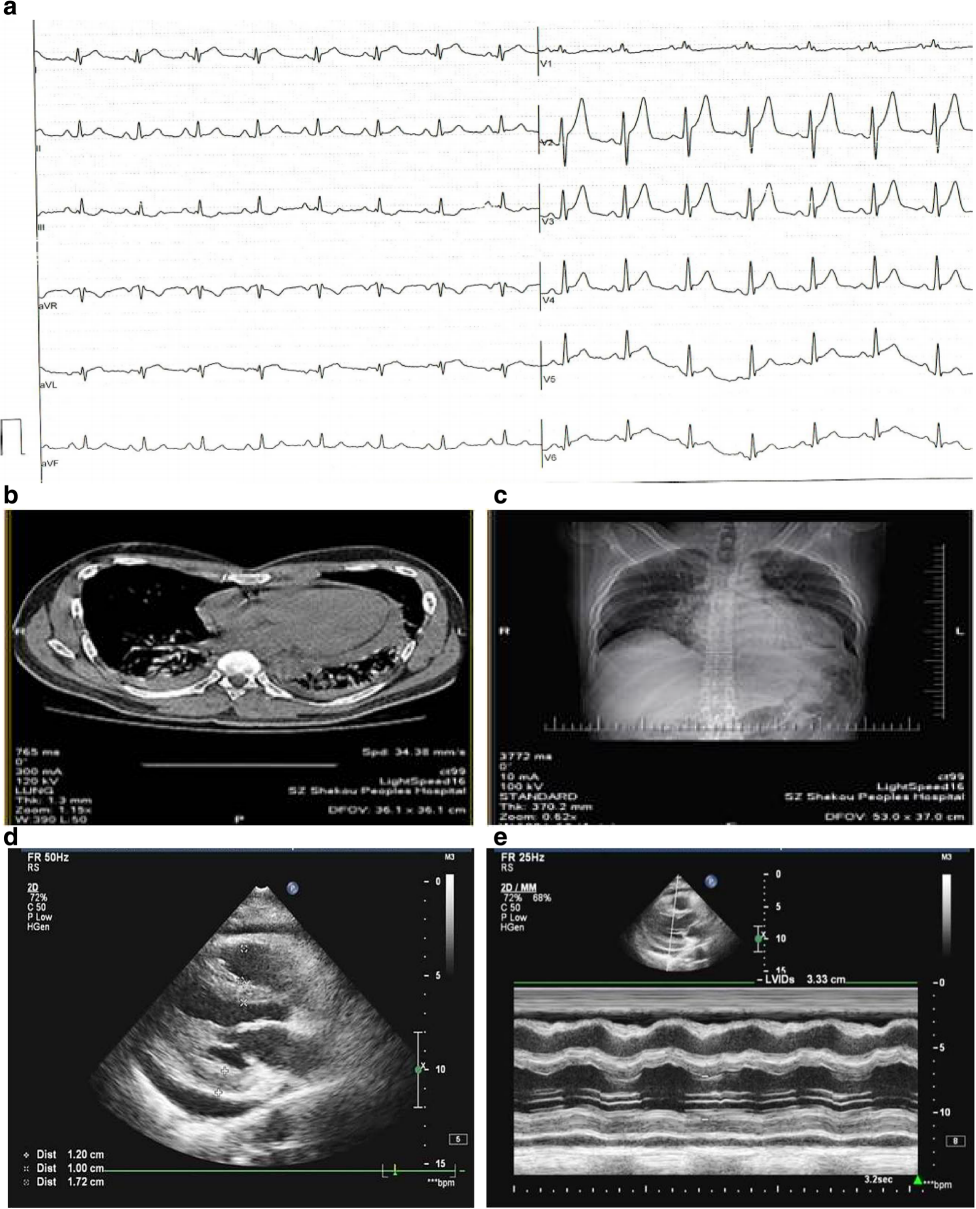
**a** After 24 h of PCI, the twelve-lead ECG showed wide spread concave ST segment elevations. **b** and **c** Chest CT scan showed mild pleural effusion and interstitial infiltration in both lungs. **d** and **e** UCG revealed mild pericardial effusion with posterior wall motion disappearance

**Fig. 3:**
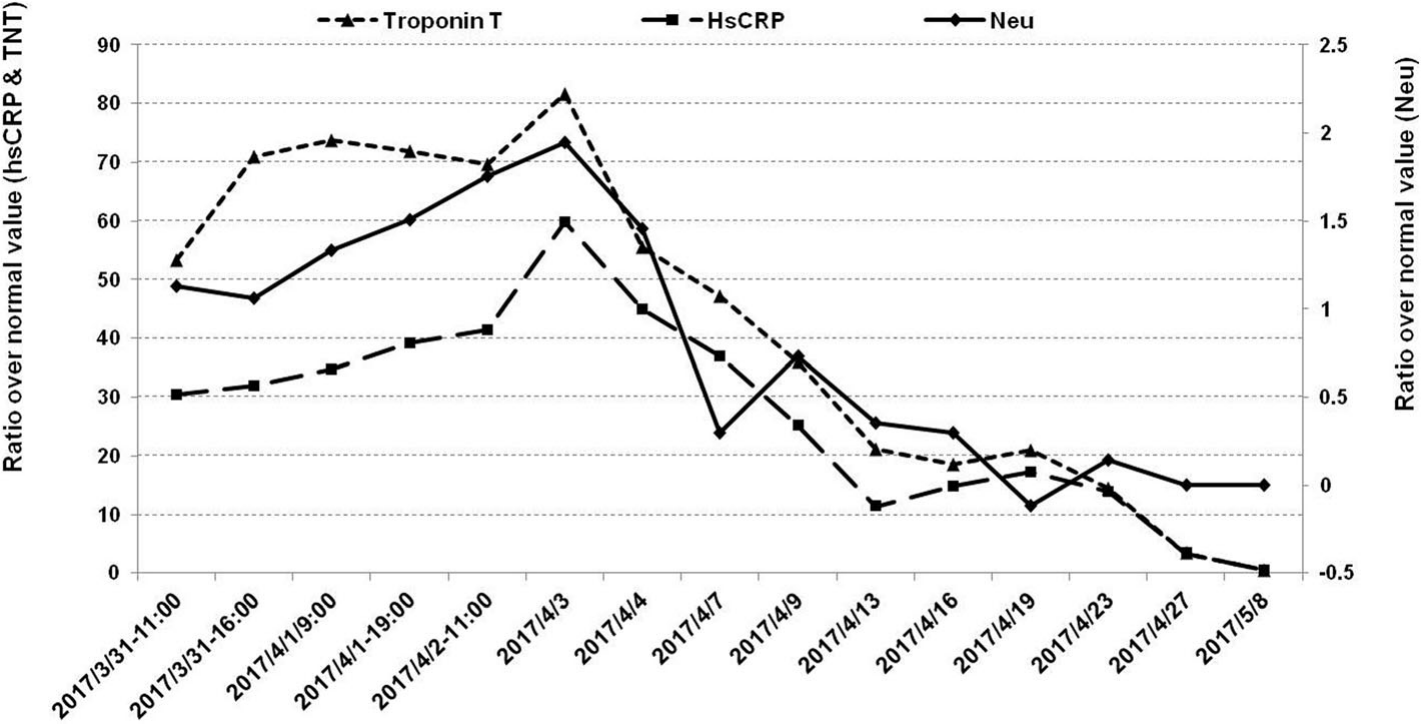
Blood test showed Neutrophil count and the serum concentration of cTnT were elevated in parallel with HsCRP increase in the early and later stage of PCIS, respectively

A 45-year-old male with known risk factors of endothelial dysfunction (including smoking and hyperlipidemia) had typical episodes of angina for 3 days. Characteristic dynamic changes of electrocardiogram (ECG) and cardiac marker of myocardial necrosis troponinT (cTnT) suggested posterior STEMI. Emergency coronary angiography (CAG) revealed complete proximal occlusion of the circumflex artery (Fig. 1a). A drug eluting stent was deployed to the proximal left circumflex artery (p-LCX). Final angiogram revealed that the PPCI was successful (Fig. 1b). Three hours later, the patient developed dyspnea and persistent pleural chest pain, and the ECG showed widespread concave ST segment elevations and PR segment depression (Fig. 2a). A follow-up CAG was performed 33 h after PPCI, and no stent thrombosis or any significant evidence of iatrogenic trauma due to the intervention procedures was found. But a significant slower TIMI flow (grade ≤ 2 grade) (Fig. 1c) and abnormal TIMI myocardial perfusion frame count (TMPFC = 140 frames, at a filming rate of 30 frames/sec.) in the culprit arteries were seen through CAG. Consistent ST segment elevation on ECG with an increase in cTnT, but no recurrent CK-MB peak, seemed to suggest that the persistent focal myocardial injury might possibly involve coronary microvascular dysfunction (CMD). In recent years, assessing coronary flow reserve (CFR) by intracoronary Doppler guide wire and positron emission tomography (PET) is considered the gold standard for quantitative assessment of coronary microcirculation disorder. But this method is technically complex and very expensive, and therefore not applicable to the present case based on the patient’s condition and intention. Chest CT scan showed mild pleural effusion and interstitial infiltration in both lungs (Fig. 2b, c), and UCG revealed mild pericardial effusion with posterior wall motion disappearance (Fig. 2d, e). Blood test showed that the serum concentration of HsCRP was persistently increasing; neutrophil count and the level of cTnT were elevated in parallel with HsCRP increase in the early and later stage of PCIS, respectively (Fig. 3). The erythrocyte sedimentation rate (ESR) (83 m/s) was also significantly elevated as another inflammatory marker, while the concentration of Anti-Streptolysin O (ASO) and Antinuclear Antibody (ANA) associated with rheumatic and tuberculosis disease and B-type natriuretic peptide (BNP 107 pg/ml) was still in the normal range. So we concluded that the patient had developed PCIS. After receiving full anti-ischemic drug treatment and aspirin at an anti-inflammatory dose, the patient was symptom-free during hospitalization. The pericardial effusion was gradually resolved along with the recovery of serum concentration of HsCRP and cTnT to the normal levels at 3 weeks after PPCI.

## Discussion and conclusions

PICS has been used to describe early-onset pleuropericarditis after AMI and coronary catheterization. The former is also called Dressler syndrome, the onset of which usually occurs 3–4 weeks after AMI. A widely accepted hypothesis about this syndrome is that an autoimmune response against heart antigens leads to generalized pericardial inflammation and pericarditis [2]. In the era of PPCI, the incidence of Dressler syndrome and PCI-related PCIS has been decreasing. Although the pathogenesis remains generally uncertain, there has been some presumption that an autoimmune pathogenesis triggered by myocardial necrosis already exists before PPCI [3] and that iatrogenic trauma may be done to the pericardial and/or pleural mesothelial cells [4]. Here we report a rare case of PCIS with CMD following PPCI. Similar to the case reported by Jin-Seok Park in 2010 [5], the PCIS occurring in our case after PCI also had an atypical early onset, and the clinical features included pleuritic chest pain, low fever, pericardial and/or pleural effusions.

Furthermore, the present case seemed to suggest that the increase of inflammatory marker in AMI patients with PCIS had potential association with PCI-related CMD and the subsequent persistent focal myocardial injury. According to the evidences from animal models [6] and clinical study [7], cardiac neutrophil activation along with the release of proinflammatory cytokines will cause damage to the microvascular endothelial and pericardial/pleural mesothelial cells. And this may finally result in CMD and pleural pericarditis along with myocardial ischemia and reperfusion. In addition, Frohlich GM et al. [8] and Robbers LF et al. [9] have also demonstrated recently that inflammatory responses due to perfusion injury stimulates the production of chemokines, cytokines, and adhesion molecules, which is probably associated with CMD and coronary microvascular obstruction. This further contributes to the development of myocardial no-reflow and myocardial hematoma. According to the report of Jin-Seok Park et al. [5], in a patient with PCIS after a successful PCI, there were some red blood cells present in the drained pericardial effusion. This suggested that the myocardial hematoma due to PCI-related CMD is likely to cause leakage of the blood into the pericardial space and thus plays an important role in the pathogenesis of PCIS.

The risk factors of microcirculatory dysfunction included smoking, obesity, hyperlipidemia, hyperuricemia, diabetes and chronic kidney disease (CKD) besides AMI. It has been found that the long-term present of stimulating signals which can induce endothelial injury may mediate the DNA methylation of chromosomes, leading to changes in chromosomal structure and function and enhancing the regulatory effect of stress signals on the signaling pathways related to endothelial injury [10]. Chromosome remodeling of endothelial cells may be caused by long-term smoking of this patient, who was thus susceptible to endothelial damage with a “metabolic memory effect”. The above situation can enhance the I/R-related endothelial damage after PCI, resulting in severe impairment of coronary microcirculatory structure and coronary flow reserve/diastolic function. It has been reported by Matteo et al. [11] that CKD can promote microcirculatory dysfunction and lead to a decrease in diastolic dysfunction and cardiac reserve function impairment. Our patient showed a persistent increase of cTnT even after the infarct-related artery (IRA) was opened by PCI. This possibly resulted from myocardial focal necrosis caused by cigarette smoking-aggravated microcirculatory dysfunction and a decrease in myocardial blood flow perfusion.

Besides, in the present case, the level of inflammatory marker also matched up with the consistent elevation of cTnT, which was probably associated with the persistent focal myocardial injury due to CMD. This agrees with the recent studies reported by Vilahur G et al. [12], who showed that extensive necrosis of ischemic cardiomyocytes in hypoperfused myocardium might activate the innate immune response, triggering an increase of compensatory inflammation. At present,the correlation between perioperative PCIS and CMD  in AMI needs to be further explored.

In conclusions, findings in the present case seemed to suggest that excessive inflammatory reaction and CMD triggered by PCI-related ischemia-reperfusion injury may be the potential reciprocal causation of PCIS. So we should pay more attention to the severe inflammatory reaction and CMD resulting from cardiac ischemia-reperfusion injury in an AMI patient with risk factors of endothelial dysfunction, although this condition poses a real challenge, especially in the PCIS-predisposed individuals.

## Abbreviations

ANA: Antinuclear Antibody
ASO: Anti-Streptolysin O
BNP: Natriuretic peptide
CAG: Coronary angiography
CFR: Coronary flow reserve
CMD: Coronary microvascular dysfunction
ESR: Erythrocyte sedimentation rate
hs-CRP: Hypersensitive C-reactive protein
PCIS: Post-cardiac injury syndrome
PET: Positron emission tomography
p-LCX: Proximal of left circumflex artery
PPCI: Primary percutaneous coronary intervention
TIMI: Thrombolysis in myocardial infarction
TNT: Troponin T
